# Robust electric-field tunable opto-electrical behavior in Pt-NiO-Pt planar structures

**DOI:** 10.1038/srep28007

**Published:** 2016-06-13

**Authors:** A. Rebello, A. O. Adeyeye

**Affiliations:** 1 Information Storage Materials Laboratory, Department of Electrical and Computer Engineering, National University of Singapore, 117576 Singapore

## Abstract

Capacitor-like metal-NiO-metal structures have attracted large interest in non-volatile memory applications based on electric field control of resistance, known as resistive switching (RS). Formation of conducting nanofilaments by the application of an electric field (electroformation) is considered an important pre-requisite for RS. Besides RS, due to the wide band gap and p-type semiconducting nature, NiO has been used to fabricate heterojunctions for photodetector applications. However, very little is known about the electrical and opto-electrical properties of NiO films in planar structure. Here, we demonstrate intriguing photoresponse and electrical behavior in electroformed Pt-NiO-Pt planar structures. While the pristine devices show ohmic electrical behavior and negligible photoresponse, the electroformed devices exhibit a nonlinear rectification behavior and a remarkable photoresponse at low voltage biases. More interestingly, the devices show a dramatic change of sign of rectification under light illumination at higher voltage biases. A polarity dependent and robust gain phenomenon is demonstrated in these devices. The large sensitivity, fast response, simple design and ease of preparation of these planar structures make them attractive for integration with current circuit technologies and various novel opto-electrical applications.

Physics of light and development of new light based technologies have attracted tremendous research interest owing to their fundamental and technological significance. For instance, photodetectors with low dark current, fast response and high sensitivity have wide range of applications such as optoelectronic devices, biomedical imaging, optical communications, quantum information technology and remote sensing^1^,^2^. Transition of electrons between different energy levels by light absorption is the fundamental operating mechanism of a photodetector. Among the commercially available photodetectors, photomultiplier tube and avalanche photodiode stand out owing to their high sensitivity and fast response^3^. However, the prominent demerits of these detectors are fragility, cost, and bulkiness. In addition, the requirement of high electric bias, additional supply voltage stabilization circuitry and intricate temperature compensation hampers the integration of these detectors into the current circuit technologies such as complementary metal-oxide semiconductor (CMOS) electronics. As an alternative, extensive efforts are underway to develop photodetectors from 1D nanowires, quantum dots, metal oxide nanorods and heterojunctions made of nanocomposites and oxide thin films^4^,^5^,^6^,^7^,^8^,^9^. Heterojunctions made of ZnO nanorods and NiO thin films have been demonstrated to exhibit modest photodetector characteristics because of a very large response time and high dark current, however, photoresponse properties of NiO thin films in the planar structure are not known to date.

NiO is a well-known p-type semiconductor with a wide band gap (~3.8 eV), and has been widely investigated for different technological applications including nonvolatile memory devices^10^, super capacitor electrodes^11^, electrochromic devices^12^, gas sensors^13^, and photocathodes for solar cells^14^. In particular, capacitor-like metal-NiO-metal structures have attracted considerable attention in the past decade due to its potential as an ultra-high density and high speed nonvolatile memory based on reproducible electric field induced changes in the resistance of a material, known as resistive switching^15^,^16^. Typically, an initial step known as electroformation is employed to the as prepared devices to facilitate resistive switching^17^. This pre-requisite involves the application of a sufficiently large electric field (>10^6^ V/cm) to the pristine devices, which creates conducting nanofilaments in the insulating host matrix^18^. Besides capacitor-like structures, electroformation on single crystalline oxide heterostructured nanowires has also been demonstrated to exhibit resistive switching characteristics^19^. Here, we present intriguing electrical characteristics and unusual photoresponse in a Pt-NiO-Pt device in in-plane geometry, which is electroformed by the application of an electric field of the order of ~10^4^ V/cm. While the pristine devices showed ohmic behavior and negligible photoresponse, a very robust nonlinear rectification behavior and photoresponse are observed in the electroformed devices. Furthermore, the devices exhibit low dark current, low response time and good responsivity at low voltage biases, which are essential qualities of a good photodetector and they can be easily integrated in to the current CMOS circuit technologies. Under light illumination at a higher voltage bias, the device shows a dramatic change in the sign of rectification, and exhibits superior photoresponse and sensitivity, which may be exploited for novel electro-optical devices and photodetection applications.

## Results

**Figure 1:**
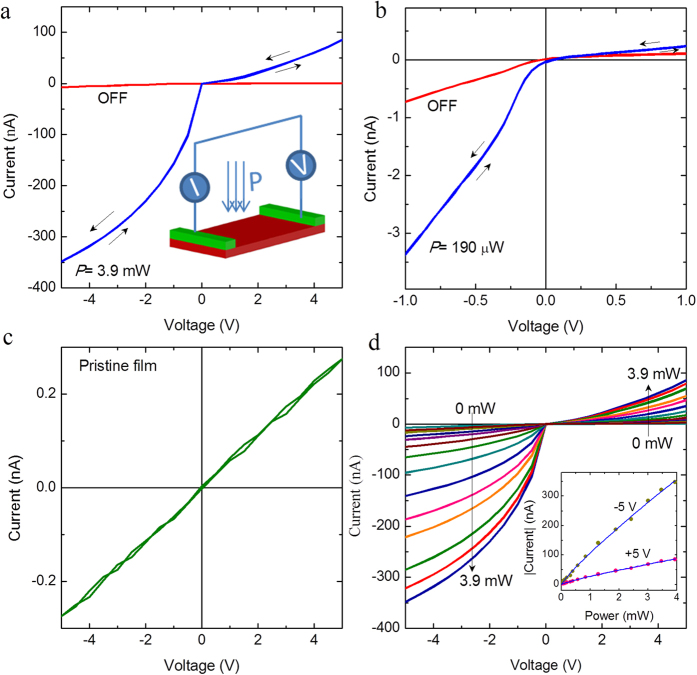
Electrical characteristics and photoresponse of in-plane Pt-NiO-Pt device. (**a**) Current *vs*. voltage characteristics of the electroformed NiO film when voltage is swept from 0 → 5 → −5 → 0 V in dark (OFF) and under visible light illumination of power, *P* = 3.9 mW. The inset shows the schematic of the measurement setup. (**b**) Current *vs.* voltage characteristics when voltage is swept from 0 → +1 → −1 → 0 V in dark (OFF) and under low power light illumination. (**c**) Ohmic current *vs*. voltage characteristics of the pristine film. (**d**) Current *vs*. voltage characteristics with increasing power of illumination from 0 to 3.9 mW. The inset shows the variation of photocurrent (*I*) at ±5 V with increasing power (*P*) of illumination. Experimental data are denoted by symbols and the lines are power law fit (*I* ~ *P*^θ^).

Figure 1a shows the current *vs.* voltage characteristics of the electroformed Pt-NiO-Pt device (device A) in dark (OFF) and under light illumination (*P* = 3.9 mW). A large enhancement in the magnitude of the current is observed from very low voltage magnitudes under light illumination. In Fig. 1b, we show the current-voltage characteristics of the electroformed device at lower voltage magnitudes in dark and under a low power light illumination (*P* = 190 μW). Another remarkable feature of the electroformed device is the presence of nonlinearity and rectification in the current-voltage characteristics. The typical current-voltage curve of an as prepared device is plotted in Fig. 1c. In contrast to the electroformed films, the current-voltage curve of the as prepared film did not show any nonlinearity and rectification. In Fig. 1d, we demonstrate the systematic variation of current *vs*. voltage curves with increasing power of light illumination, when voltage is swept from 0 → 5 → −5 → 0 V. The photoresponse under negative voltage bias is larger compared to the forward bias at all powers of illumination. Interestingly, the curves do not exhibit any hysteresis behavior, which favors its practical implementation for device applications. The variation of the magnitude of current (*I*) at ±5 V is plotted as a function of illumination power (*P*) in the inset of Fig. 1d. The solid lines are power law (*I* ~ *P*^θ^) fit to experimental data (symbols). The fitting gives a non-linear behavior with θ = 0.85. The non-unity (0.5 < θ<1) exponent suggests the presence of traps that have a distribution in energy to favor a complex process of electron–hole generation, recombination, and trapping within the semiconductor^20^. Under electronic doping from light excitation, many trapping states will be converted to recombination states. This increases the number of recombination states for electrons, which, in turn, reduces the electron lifetime and therefore, the value of θ deviates from unity.

**Figure 2:**
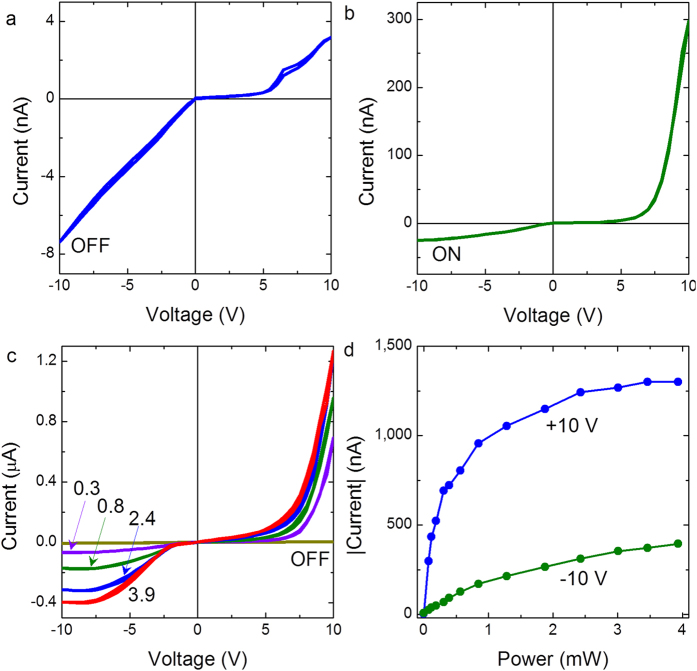
Effect of light on electrical asymmetry at high voltage bias. Current *vs*. voltage characteristics of the NiO film when voltage is swept from 0 → +10 → −10 → 0 V in (**a**) dark (OFF) and (**b**) under 75 μW power of light illumination (ON). (**c**) Current vs. voltage characteristics under different powers of light illumination, the numbers denote the magnitude of power in milliwatts. (**d**) Photo current *vs.* power of illumination at ±10 V.

Motivated by the large photoresponse at low bias voltages, we further investigated the current-voltage characteristics of the device A at higher voltage magnitudes. Figure 2a shows the current-voltage curve when the voltage is swept from 0 → 10 → −10 → 0 V under dark ambience and Fig. 2b, under a very low power of light illumination *P* = 75 μW. Surprisingly, the magnitude of current under light illumination exhibited a large enhancement under positive voltage bias compared to the negative bias, resulting in a dramatic change in the sign of rectification. Note that this is in sharp contrast to the electrical behavior at low voltage magnitudes (Fig. 1d), where the photoresponse was larger under negative bias. In addition, the current-voltage curves showed a systematic variation under varying power of light illumination, as illustrated in Fig. 2c and the curves under light illumination were highly reproducible and robust. The variation of current as a function of illumination power at ±10 V is plotted in Fig. 2d.

**Figure 3:**
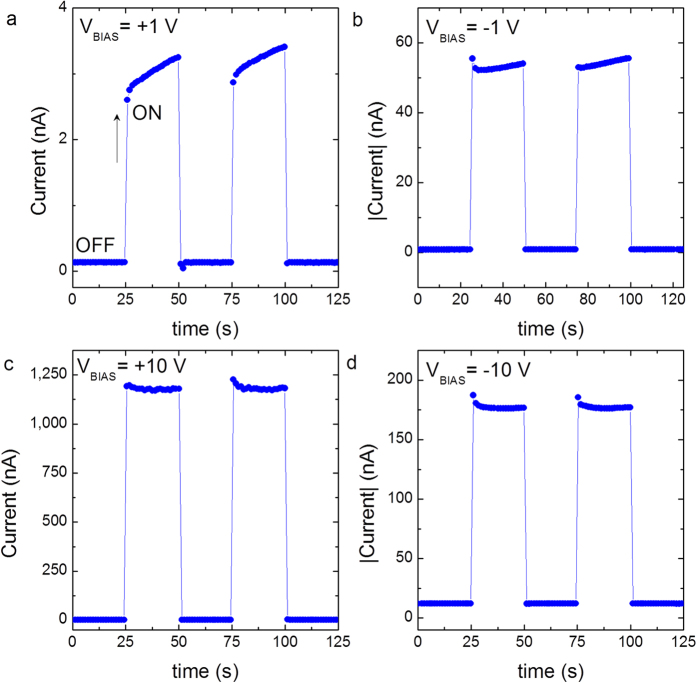
Reproducible photodetection of the device. The variation of photocurrent when light is switched ON and OFF at regular interval of 25 s at various bias voltages (**a**) +1 V, (**b**) −1 V, (**c**) +10 V and (**d**) −10 V.

The photoresponse of the device at low (±1 V) and high (±10 V) voltage biases are shown in Fig. 3. In all the cases, the photocurrent can be reproducibly generated by periodically turning the light illumination on and off. The device has a very low dark current (a few nanoamperes) at all voltage biases. Upon illumination, the photocurrent rapidly increased to a stable value, and then drastically decreased to its initial level when the light was turned off, indicating the excellent stability and reproducible characteristics of the device. In close agreement with the photoresponse of the current-voltage characteristics (Fig. 2d), a large photocurrent of approximately 1100 nA is observed at +10 V with a very low dark current (a few nanoamperes) for *V*_*BIAS*_ = 10 V (Fig. 3c). Under all the voltage biases, the response time of the device was less than the 200 ms, the actual response time could not be measured in this study due to experimental limitations. The above mentioned features render the devices under present study much superior photodetection attributes compared to NiO-ZnO heterojunctions, where the response time is a few tens of seconds with high dark currents^8^,^21^. The responsivities of the photodiode obtained using a red laser beam with intensity of illumination *I* = 15 mW/cm^2^ are 0.24 AW^−1^ (+10 V) and 0.16 AW^−1^ (−1 V). Interestingly, a high responsivity of 0.25 AW^−1^ has been demonstrated in an infrared photodetector based on hot electron carrier generation^22^; however the device was cooled down to low temperatures to reduce the presence of high dark current arising from a low potential barrier. At 10 V bias, the present devices showed a large responsivity in the sensitive region (Fig. 3d), i.e., at low powers of light illumination. The responsivity of the present device is 21.7 AW^−1^ at 10 V and 46 μW/cm^2^ illumination power, which is comparable to the responsivity (3–16 AW^−1^) recently reported in germanium/silicon avalanche photodiodes above 22 V^23^. Furthermore, those diodes exhibited a higher dark current arising from the tunneling current at the silicon/germanium interface at higher voltage biases.

## Discussion

**Figure 4:**
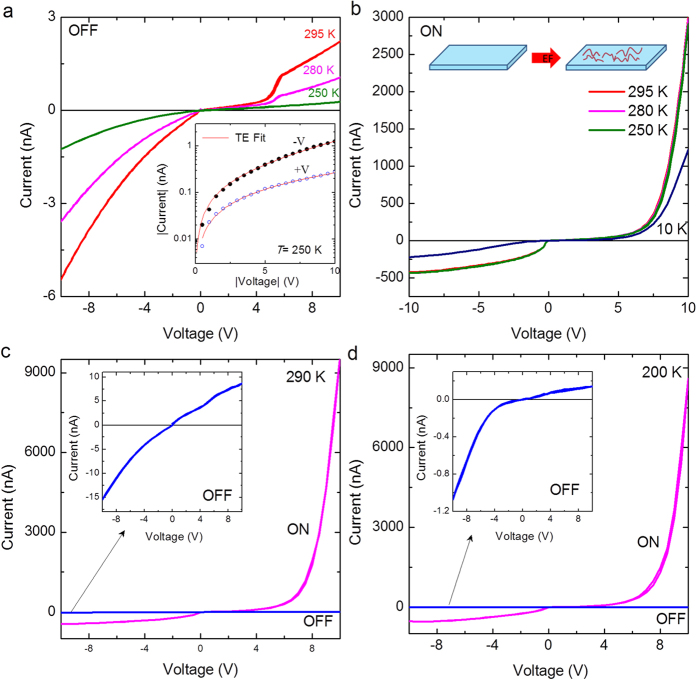
Photoresponse at different temperatures. Current *vs.* voltage characteristics of the device B at different temperatures, *T* = 295, 280 and 250 K in the (**a**) OFF and (**b**) ON state. Photoresponse of the re-electroformed device B at (**c**) *T* = 290 K and (**d**) *T* = 200 K.

Formation of an unintentional interface layer between metal-oxide interfaces during sample preparation or trapping of charges at the metal-semiconductor interface can lead to non-ohmic behavior^24^. The ohmic behavior observed in the present pristine devices suggests that the semiconductor-metal junctions are free of any interfacial capacitance. Interestingly, the electroformed device exhibits a strong nonlinearity and rectification even at lower voltage magnitudes. Ohmic contacts between metal-semiconductor junctions are achieved by high doping concentration or low barrier height^25^. The theoretical barrier height for a p-type semiconductor- metal junction is defined as φ_B_ = *E*_*g*_/*q* + *χ*−φ_m_, where *E*_*g*_ is the band-gap, *χ* the electron affinity of the semiconductor and φ_m_ the work function of metal. For NiO-Pt junction, *E*_*g*_ ~3.8 eV^26^, *χ* *=* 1.46 eV^27^, φ_Pt_ = 5.4 eV, which yields a low theoretical barrier height ~0.1 eV. Therefore, the non-ohmic nature of the electroformed Pt-NiO-Pt devices implies the formation of Schottky barriers at the metal-oxide interfaces during electroformation. To investigate the Schottky characteristics, we further performed photoresponse measurements in a similar device (named as device B) at different temperatures, *T* = 295, 280, 250 and 10 K. The current-voltage characteristics under dark ambience (OFF) and light illumination (ON) are shown in Fig. 4a,b, respectively. In contrast to the current-voltage curves in the ON state, the curves in the OFF state showed prominent variation. Notably, the prominent kink-like behavior observed around 5 V at 295 K in the OFF state is absent at 250 K (Fig. 4a). In addition, the device showed considerable photoresponse even at 10 K (Fig. 4b), whereas, the *I*-*V* characteristics under dark ambience was too low to be measured at 10 K. In general, current-voltage characteristics of Schottky junctions can be described by the thermionic emission theory:
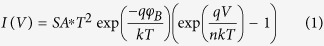
where *S* is the junction area, A^*^ is the Richardson constant, *q* is the electron charge, *k* is the Boltzmann constant, *T* is the temperature, *n* is the ideality factor and 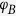 is the Schottky barrier height. While the value of *n* in ideal thermionic emission is equal to unity^28^, theoretical fits to the experimental data at 250 K in the OFF state yielded high values of *n* (>300) (inset of Fig. 4a), which indicates the absence of an ideal Schottky barrier scenario. However, the experimental data in the ON state could not be reproduced by the TE theory. To further study the dependence of optoelectrical response on the Schottky characteristics and stochastic nature of electroformation, electroformation was re-performed on device B. The photoresponse of current-voltage characteristics at 290 K in the re-electroformed device B is shown in Fig. 4c. Interestingly, the re-electroformed device B in the OFF state showed poor rectification (inset of Fig. 4c) and did not exhibit any prominent kink, unlike the previous data (Fig. 4a). However, at *T* = 200 K, the device showed a prominent rectification in the OFF state (inset of Fig. 4d). Though the device showed a poor rectification ratio at 290 K compared to that at 200 K under dark ambience, it showed a large and comparable photoresponse in the ON state at 290 K (Fig. 4c) and 200 K (Fig. 4d). This further corroborates that the degree of rectification is not a significant aspect determining the optoelectrical response. Therefore, we consider another scenario based on electroformation, to explain the intriguing optoelectrical and nonlinear response in the electroformed device, wherein the electroformed oxide layer comprises of nanofilaments formed by the application of high electric field^16^,^29^,^30^,^31^. A schematic of the electroformed device is shown in the inset of Fig. 4b. During electroformation, it is highly likely that Ni-O bonds can break and oxygen atoms escape from the oxide layer. The formation of oxygen vacancies and metallic Ni states leads to a change in the resistance of the device. These conducting nanofilaments act as defects modifying the electrical properties and band structure of the pristine device. This is corroborated by the low resistance of the electroformed devices compared to the pristine devices (lower by one order of magnitude in the present study) and the nonlinear nature. Note that a large nonlinear asymmetric current-voltage behavior has been demonstrated in capacitor-like structures based on NiO nanoparticle assembly^32^. The presence of random dopants and local variation in chemical composition can cause potential fluctuations, leading to local variation of the semiconductor band edges and bandgap energy fluctuations^33^.While the pristine device is unresponsive to the light radiation, the electroformed device shows strong response to visible light and red laser beam (λ = 650 nm). Since the cut-off wavelength calculated using NiO bandgap (3.6 eV) is λ_c_ = *hc*/*E*_*g*_≈ 343 nm, it is interesting that the electroformed device shows photoresponse even at higher wavelength. This strongly suggests that the band structure of the oxide layer in the pristine device is completely different from that of the electroformed device. It is well known that defects in NiO play a significant role in determining the band structure and the conductivity. Recently, Peng *et al*. has demonstrated that measurement protocols such as current compliance and method of sample preparation can control the stoichiometry and the associated defect characteristics, which in turn modifies the band structure and leads to different electrical characteristics in a NiO based device^34^. Based on the observation of decrease in the resistivity of the electroformed device in the present study, we infer that electroformation has modified the defect characteristics of the pristine oxide layer, which is supposed to be a charge transfer insulator in its purest form.

**Figure 5:**
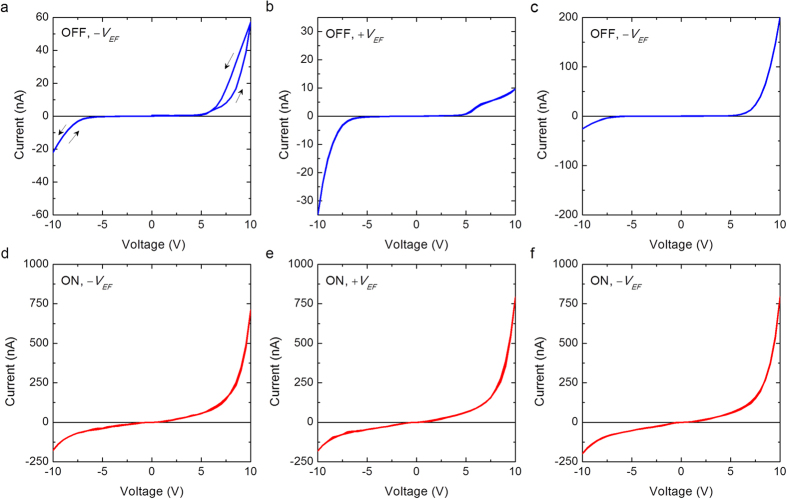
Influence of bias direction of electroformation on electrical behavior. Current *vs*. voltage characteristics in the OFF and ON state of an electroformed device, when subsequently re-electroformed with negative forming voltage −*V*_*EF*_ [(**a**,**d**)], positive forming voltage +*V*_*EF*_ [(**b,e**)], and −*V*_*EF*_ [(**c**,**f**)]. Note the toggling of direction of rectification in the OFF state with the forming bias direction, whereas the electrical behavior in the ON state shows negligible change.

In the following, we demonstrate that electroformation is a key factor in determining the electrical behavior of the devices in the present study. Figure 5a shows the nonlinear and asymmetrical electrical behavior in the OFF state of a device when re-electroformed by changing the bias direction of the forming voltage (*V*_*EF*_) to negative polarity. Interestingly, the direction of rectification is reversed; please see the typical rectification behavior when electroformed with positive bias direction (Fig. 2a) for comparison. Figure 5b,c shows the rectification behavior in the OFF state when the device was subsequently electroformed with positive and negative voltage polarities, respectively. The toggling of direction of rectification behavior in the OFF state with the bias direction of forming electric field confirms the impact of electroformation on the rectification behavior. On the contrary, the change of bias direction of electroformation showed negligible impact on the current-voltage behavior in the ON state, as can be seen in Fig. 5d–f.

The devices exhibited a robust photoconductivity with low dark current levels and did not suffer from any persistent photocurrent problems and degradation of the photoconduction quality by over exposure to light. Note that in GaN-based photoconductors, the photocurrent persists for a long time (hours) after the light is shut off and the response speed for the photoconductor is very slow, hampering its implementation in real device applications^35^,^36^. In contrast to the photoresponse at low voltage magnitudes (inset of Fig. 1d), photocurrent *vs.* power for both positive and negative 10 V biases (Fig. 2d) could not be fitted to any power law. The origin of the dramatic enhancement in the photocurrent and sensitivity at higher voltages may be different from the usual electron–hole generation, recombination, and trapping within the semiconductor. We note that the planar structure of the present device is similar to the geometrical configuration of a metal-semiconductor-metal (MSM) photodiode and the increasing photocurrent with increasing voltage above ~5 V under positive voltage bias closely resembles the gain phenomena in a MSM photodiode^37^. MSM photodetectors generally consist of two Schottky contacts, in the form of interdigitated fingers deposited on a low-doped semiconductor, that act like two back to back Schottky diodes and exhibiting a nonlinear but symmetric behavior in the OFF state^38^. However, the present device exhibits an asymmetric nonlinear behavior which varies depending on the stochastic nature of electroformation and formation of conducting nanofilaments. While MSM based photodetectors requires a high voltage bias for a reasonable photoresponse, the present device shows remarkable photoresponse at very low magnitude of voltage bias, which further indicates that the fundamental mechanism of MSM photodiode is different from that of the present device. Theoretical fits based on various interface and bulk limited conduction mechanism did not yield good match with the experimental data under light illumination. The exact microscopic mechanism for the increase in the photocurrent under high positive voltage bias is unclear and therefore calls for further theoretical and experimental investigations. The increasing photocurrent with increasing voltage in the present device can be qualitatively explained by considering holes or electrons trapped at the surface near electrodes, which creates an asymmetric electric field at the metal-semiconductor contact. Under sufficiently high voltage bias, an additional carrier injection is induced from the electrodes by tunneling, leading to an enhanced photocurrent. Another plausible scenario is the impact ionization generated at high voltages similar to an avalanche photodetector^39^,^40^. We note that the robust and reproducible opto-electric response in our devices deteriorated when they were operated above 15 V, possibly due to a soft-breakdown occurring in the oxide layer. Furthermore, surface sensitive spectroscopic studies such as x-ray absorption spectroscopy or electron energy loss spectroscopy, which is beyond the scope of present work, can shed more light on the chemical nature of the electroformed oxide layer in the pristine device.

In summary, we have studied opto-electrical properties of planar Pt-NiO-Pt device. While the pristine device shows ohmic behavior and negligible photoresponse, this study demonstrates that an electroformed device exhibits a remarkable photoresponse and asymmetric nonlinear electrical characteristics. At lower voltage magnitudes, the device shows a larger photoresponse under negative voltage bias compared to that under positive voltage bias. In sharp contrast, at higher voltage bias, the device shows an excellent sensitivity to light illumination under positive voltage bias, manifested as a dramatic reversal of asymmetry in the electrical characteristics. The ease of fabrication, robustness and the simple design of the current devices facilitate the exploitation of the observed photoresponse behavior and the monolithic integration of these structures with current circuit technologies for a variety of light-based applications.

## Methods

Pt -NiO-Pt devices (schematic is shown in Fig. 1) were prepared on a SiO_2_/Si substrate using standard photolithography, rf-sputtering and lift-off method. The NiO film (50 nm thickness) was deposited on 150 μm × 500 μm patterned area on the substrate at room temperature using a NiO (99.99%) target at an rf power of 100 W, 3 × 10^−3^ Torr working pressure and in deposition chamber with a base pressure of less than 3 × 10^−8^ Torr. Two 200 nm thickness Pt electrodes, separated by 200 μm were deposited on the top surface of NiO films. A single irreversible forming by the application of an electric field of the order of 10^4^ V/cm (400–500 V) for five to eight minutes was essential for the devices to show robust photoresponse and nonlinear rectification behavior. The current-voltage characteristics were measured with two dc probes (on xyz stages) using a picoammeter (Keithley, 6487) in the in-plane geometry. The device characteristics at cryogenic temperatures were performed in a Lakeshore cryogenic probe station. A Bruker X-ray diffractometer in grazing incidence mode was used to confirm the phase of the deposited NiO films. The photoresponse measurements were performed using a quartz halogen fiber optic illuminator under dark ambience, as in a black body experiment.

## Additional Information

**How to cite this article**: Rebello, A. and Adeyeye, A. O. Robust electric-field tunable opto-electrical behavior in Pt-NiO-Pt planar structures. *Sci. Rep.* **6**, 28007; doi: 10.1038/srep28007 (2016).
